# Supplementary material for: Photoelectron circular dichroism in angle-resolved photoemission from liquid fenchone
Source: Phys Chem Chem Phys. 2022 Feb 18;24(14):8081–92. doi: 10.1039/d1cp05748k (PMC8985659; doi:10.1039/d1cp05748k)
Supplement: CP-024-D1CP05748K-s001 [file CP-024-D1CP05748K-s001.pdf]

Electronic Supplementary Material (ESI) for Physical Chemistry Chemical Physics.
This journal is © the Owner Societies 2022

Photoelectron circular dichroism in angle-resolved photoemission from liquid fenchone: Electronic Supplementary Information

Marvin Pohl,[a,b,c,‡] Sebastian Malerz,[a,‡] Florian Trinter,[a,d] Chin Lee,[b,c] Claudia Kolbeck,[a,§] Iain Wilkinson,[e] Stephan Thürmer,[f] Daniel M. Neumark,[b,c] Laurent Nahon,[g] Ivan Powis,[h] Gerard Meijer,[a] Bernd Winter,[a] Uwe Hergenhahn[a]

1 Data analysis and quality

1.1 Typical raw data

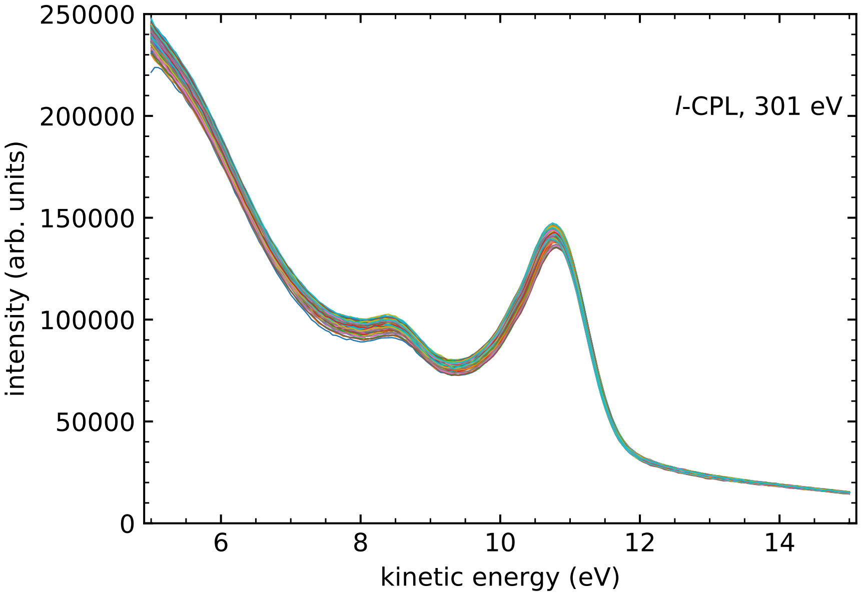

Supplementary Fig. S1 Exemplary, single-sweep photoelectron spectra recorded from liquid (1S,4R)-fenchone, ionized with photons of 301 eV, see the sweep-averaged spectrum in Fig. 1 in the main article. Spectra were acquired in series of thirty sweeps each, with a change of photon helicity after each series. Here, traces from four series, recorded with *l*-CPL are shown. The spectra overlap due to the finite plot resolution.

In supplementary Figs. S1 and S2, the properties of an exemplary set of liquid fenchone photoemission data are shown.

[a] *Molecular Physics, Fritz-Haber-Institut der Max-Planck-Gesellschaft, Faradayweg 4-6, 14195 Berlin, Germany. E-mail: hergenhahn@fhi-berlin.mpg.de*
[b] *Department of Chemistry, University of California, Berkeley, CA 94720, USA*
[c] *Chemical Sciences Division, Lawrence Berkeley National Laboratory, Berkeley, CA 94720, USA*
[d] *Institut für Kernphysik, Goethe-Universität Franfurt am Main, Max-von-Laue-Straße 1, 60438 Frankfurt am Main, Germany*
[e] *Department of Locally-Sensitive & Time-Resolved Spectroscopy, Helmholtz-Zentrum Berlin für Materialien und Energie, Hahn-Meitner-Platz 1, 14109 Berlin, Germany*
[f] *Department of Chemistry, Graduate School of Science, Kyoto University, Kitashirakawa-Oiwakecho, Sakyo-Ku, Kyoto 606-8502, Japan*
[g] *Synchrotron SOLEIL, L'Orme des Merisiers, St. Aubin, BP 48, 91192 Gif sur Yvette, France*
[h] *School of Chemistry, The University of Nottingham, University Park, Nottingham, UK*
‡ These authors contributed equally to this work.
§ Current address: sonUtec GmbH, Mittlere-Motsch-Straße 26, 96515 Sonneberg, Germany.

1.2 Temporal stability

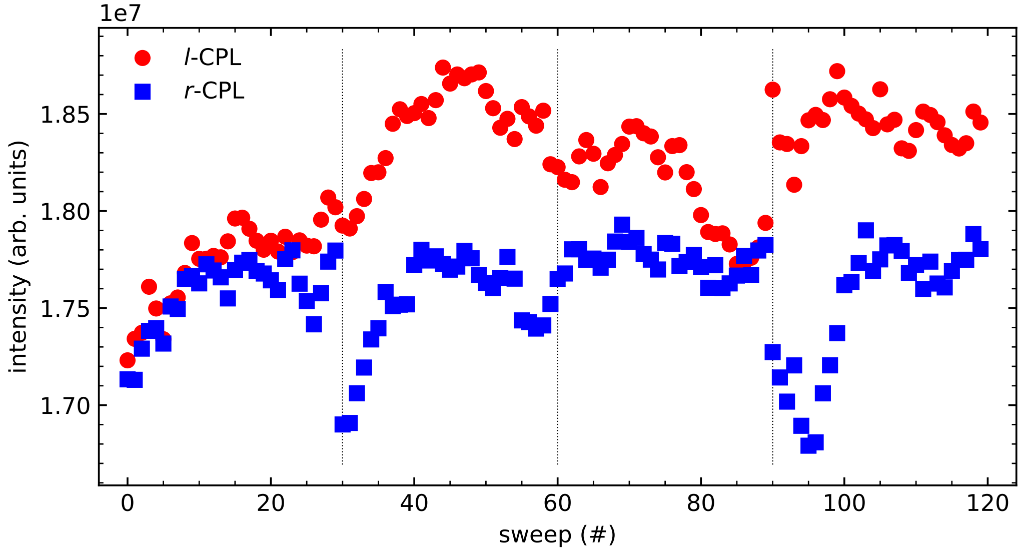

Supplementary Fig. S2 Intensity fluctuations in series of photoelectron spectra from liquid (1S,4R)-fenchone. For each sweep, we show the summed intensity in units yielded by the acquisition software of the electron analyzer. The data shown for l-CPL correspond to the sweeps shown in Supplementary Fig. S1. The vertical dotted lines indicate a shift in helicity, performed after thirty sweeps. The acquisition started by recording sweeps 0-29 with l-CPL.

Small but noticeable fluctuations of the intensity between sweeps are immediately visible in Supplementary Fig. S1. The temporal behaviour of the intensity is shown by plotting the integrated counts of every sweep in Supplementary Fig. S2. A better view of the fluctuations occurring on a short time scale is offered by plotting the point-to-point variation

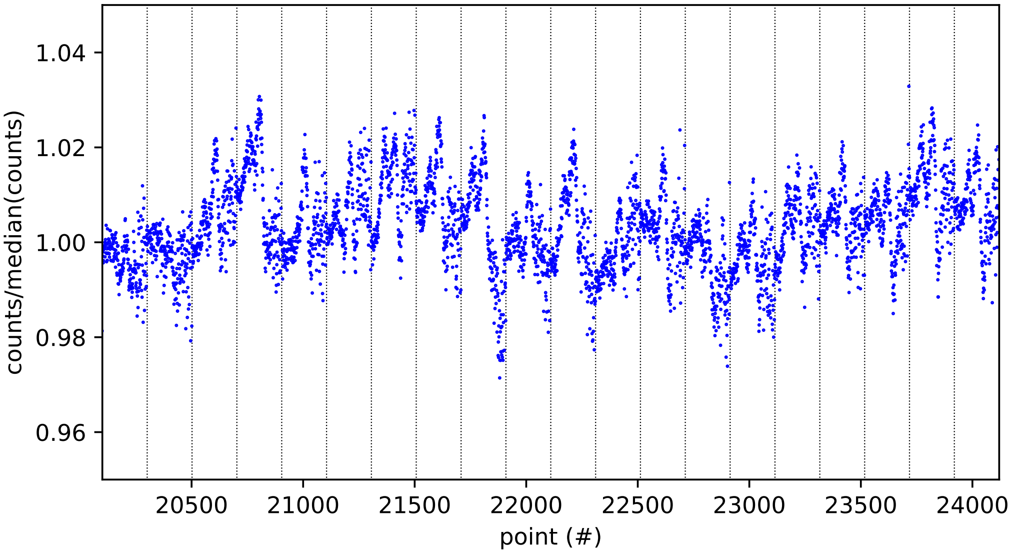

Supplementary Fig. S3 Intensity fluctuations in a series of sweeps to record the photoelectron spectrum from liquid (1S,4R)-fenchone with r-CPL. For each kinetic energy i in sweep k, we plot the counts $a_{i,k}$ divided by the median of $\{a_{i,k};\ k = 1, \ldots, 120\}$. For plotting, the points have been renumbered consecutively over all sweeps, and the dotted vertical lines show the beginning of a new sweep. A representative interval of the 120 sweeps recorded in total is shown.

of the signal intensity shown in Supplementary Fig. S3. Points were recorded with a dwell time of 0.24 s, the time interval

shown in the figure therefore corresponds to an acquisition time of approximately 1350 s, including a time margin allowed for settling of the analyzer voltages as the centrally analysed electron kinetic energy is swept.

1.3 Peak area integration, 'roi'-method

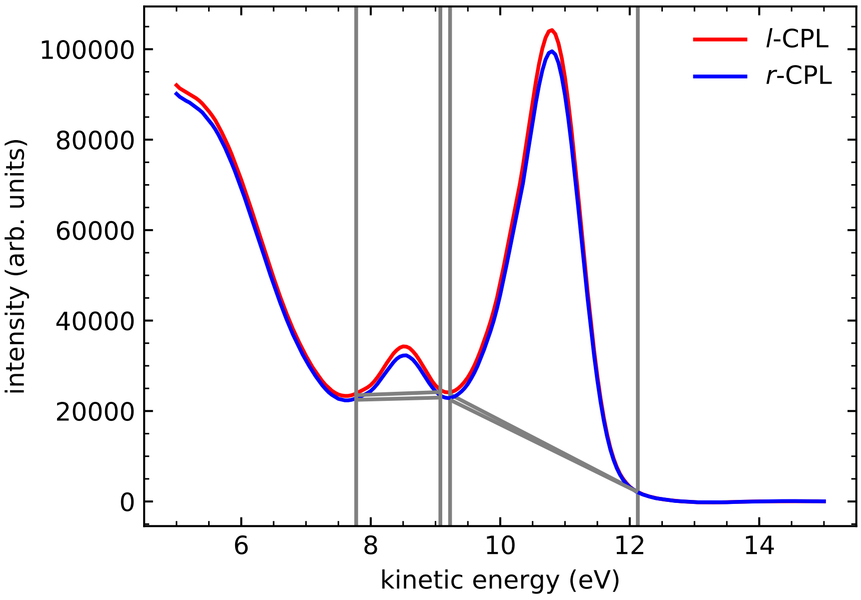

Supplementary Fig. S4 Photoelectron spectrum from liquid (1S,4R)-fenchone, ionized with a photon energy of 301 eV, after subtraction of an exponential background. For each photon helicity, the average over all sweeps is shown. The grey vertical lines mark regions used to determine the intensity of the C-H and C=O features, respectively, and the remaining grey lines indicate an additional background for each peak. Most of the apparent asymmetry between the two traces in this graph is an apparatus-induced artifact produced by a slight imbalance of photon intensity from the helical undulator in its two settings.

Here, we describe the peak-area integration method illustrated in Fig. 2A of the main text, termed the 'roi'-method. To determine areas of the two core-level ionization features, firstly an exponential background was subtracted individually from each sweep. The exponential background is determined by zeroing the higher eKE end. Averaging over the resulting data sets gives the traces shown in Supplementary Fig. S4. (The traces shown in Fig. 2A of the main article differ from the results shown in Supplementary Fig. S4 by the exclusion of a small number of outlier sweeps, and by the correction of the *l*-CPL trace for the apparatus asymmetry.) In the *ansatz* described in connection with Fig. 2A of the main text, net areas of the peak features were then determined between a low and high kinetic energy limit as indicated, subtracting a linear background. (Hence, in total, two different background contributions, a linear and an exponential one, were subtracted.) Values of the chiral asymmetry parameter, b_1, shown in the main article were determined from these net areas, correcting for the asymmetry of the C-H line.

1.4 Additional data on the peak-asymmetry analysis

Here, we provide additional evidence for the robustness of our data by showing results of a slightly more expensive analysis. From the two helicity sets of background-subtracted sweeps, we formed pairs of sweeps carried out with positive and negative photon helicity by relating them by their sweep number. (The sweeps within a pair were not measured back-to-back, as we changed photon helicity only every thirty sweeps, in this example.) For each pair, the asymmetry of the C-H feature and of the C=O feature were calculated, and the latter was corrected by requiring that the asymmetry of the C-H feature vanishes when the cumulative signal of the nine associated carbons is collectively considered. The result of this analysis is shown in Supplementary Fig. S5. Although the scatter between individual pairs of spectra is quite large, a clear trend prevails in the raw data: With very few exceptions, the uncorrected asymmetry of the C=O peak is larger than that of the C-H peak. Correction of the C=O asymmetry by the apparatus asymmetry (main text, eqn.s (1)-(3))

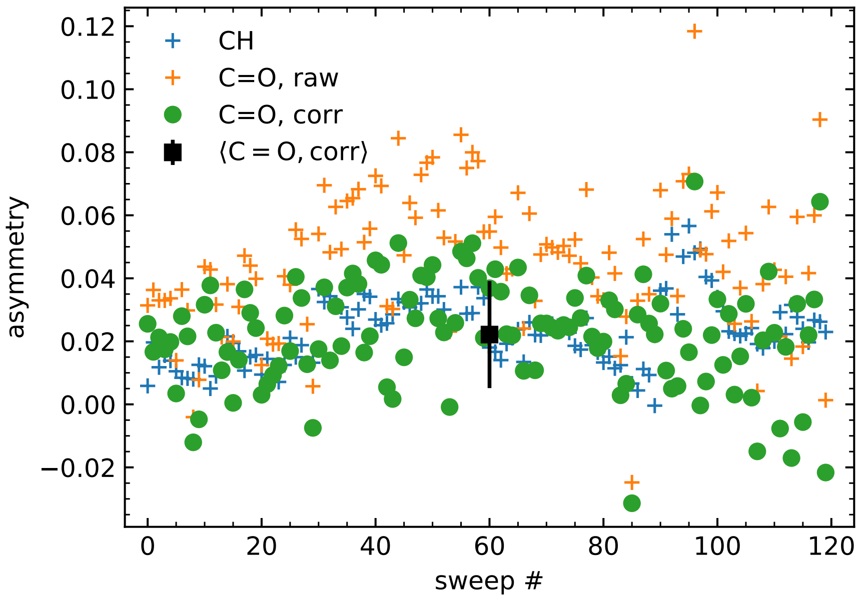

Supplementary Fig. S5 Asymmetry A and corrected asymmetry A_{corr} calculated from the individual sweeps of the data set shown in Supplementary Fig. S1. The standard deviation of the set of corrected asymmetries is shown as an error bar to illustrate the data quality. The standard deviation of the mean is smaller than the symbol size. See the text for details.

leads to smaller values of the former, which nevertheless are significantly different from zero. In Supplementary Fig. S5, the standard deviation of the data set is shown for illustrative purposes. The standard deviation of the mean, which is a more adequate measure of the actual uncertainty of the measurement, is smaller than the symbol size (exact values for the asymmetry, standard deviation, standard deviation of the mean are 0.022, 0.017, and 0.002, respectively). Supplementary Fig. S5 is based on all of the data that were recorded. In the data analysis presented in the main article, a small number of traces that deviated in shape from the majority of the recorded sweeps were removed prior to signal averaging. For this example, results of the asymmetry analysis were essentially unchanged from the value given above.

1.5 Peak area integration, 'exp'-method

For the second ansatz, highlighted in the main text Fig. 2B and labeled 'exp', we fitted a linear combination of an exponential and a linear function as a baseline for the averaged spectra. However, we only considered data points corresponding to the photoelectron background spectral regions in the fitting procedure. The included range is indicated in the top panel of Supplementary Fig. S6 by the black cross markers ('fit points') which cover background regions that are well separated from the main photoemission features. The slope by which the LET ascends towards lower energies reduces at the very-low-KE end in our spectra (possibly due to the analyzer transmission function). Therefore, it is necessary to select the 'fit points' such that the very-low-KE region is not included, specifically to guarantee that the main features do not intersect the zero of the x-axis. The resulting background-subtracted spectra are shown in the middle panel. The spectra were then normalized to the maximum C-H peak intensity and an 1.4 eV energy range was defined around the C=O peak, from which the asymmetries between different helicities were calculated. The result is displayed in the bottom panel of Supplementary Fig. S6.

1.6 Peak-area integration, 'sum'-method

The third ansatz, referred to in the main text Fig. 2C and labeled 'sum', is similar to the method discussed above in Section 1.5, with the only exception being that the background signal contribution was computed using a two-step approach. First, an exponential function was fitted to the high-KE end of the spectra, as displayed in the top panel of Supplementary Fig. S7. The resulting spectra (middle panel) where then used to perform a 'total-sum fit' (also referred to as a non-iterative Shirley method[1] or total background[2]), which iterates from the high- to the low-KE end of the spectra while

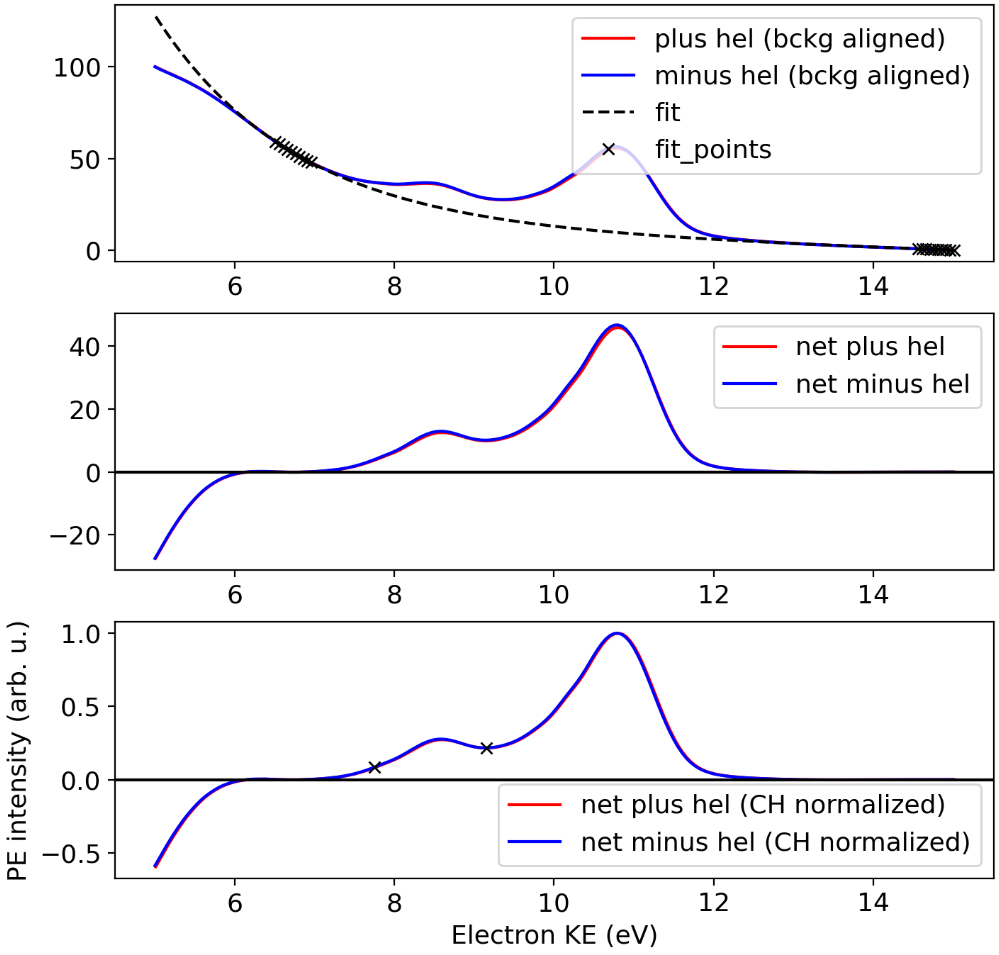

Supplementary Fig. S6 Background subtraction using the 'exp'-method for a photoelectron spectrum from liquid (1S,4R)-fenchone, ionized with a photon energy of 301 eV. Top: For each photon helicity, the average over all sweeps is shown. The dashed line shows a background fit using a combination of a linear and an exponential function. The cross marks indicate the background signal regions used for the fit. Middle: Background-subtracted spectra. Bottom: Background-subtracted spectra, normalized to the maximum CH peak intensity at about 11 eV KE. The cross marks indicate the region used to determine the intensity of the C=O features, and from that the spectral asymmetry.

aggregating spectral intensities. The correspondingly created trace was then subtracted from the middle-panel spectrum, finally producing the bottom spectrum after C-H maximum-peak normalization. Note that the so-produced background spectrum was always scaled by a factor (x 0.37 in the case of Supplementary Fig. S7), to ensure that it did not intersect with the C 1s primary photoelectron spectrum. Thus, a final spectrum was produced that intersects with the zero of the x-axis. The background scaling factor was determined iteratively such that the background touches the spectrum at a single point only.

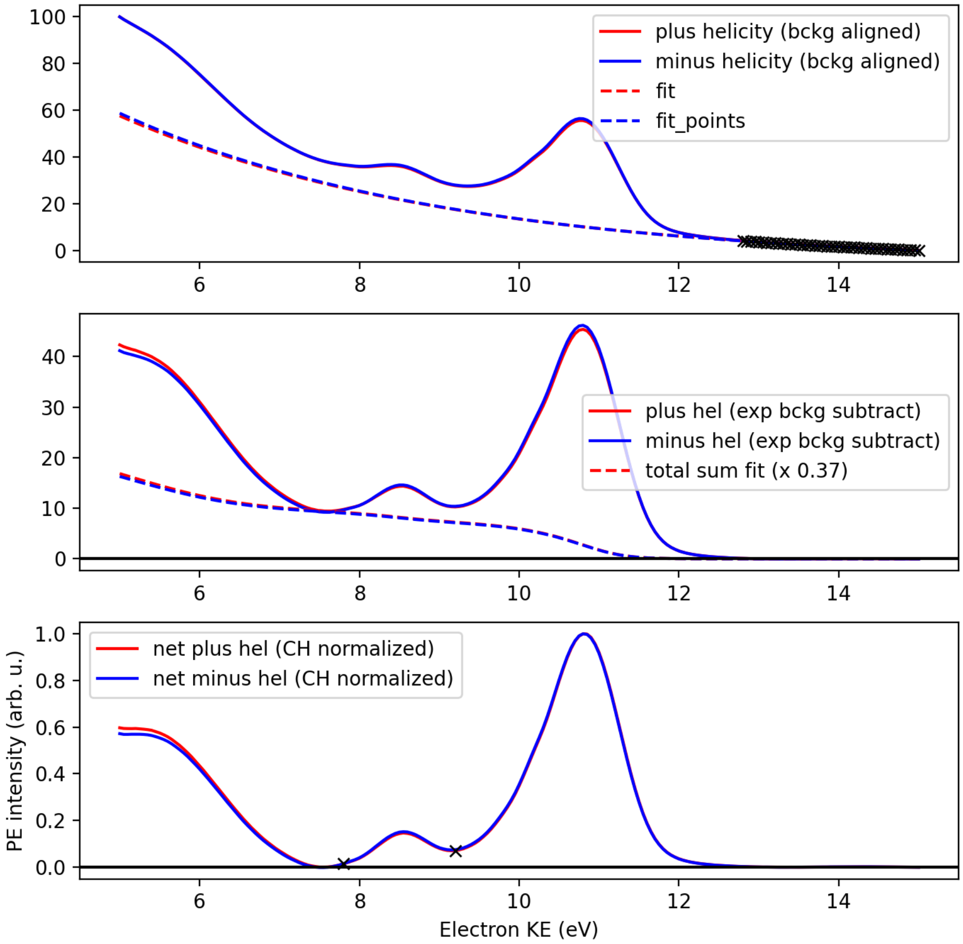

Supplementary Fig. S7 Background subtraction using the 'sum'-method for a photoelectron spectrum from liquid (1S,4R)-fenchone, ionized with a photon energy of 301 eV. Top: For each photon helicity, the average over all sweeps is shown. The dashed line shows the background fits produced using an exponential function. The cross marks indicate regions used for the fit. Middle: Exponential background-subtracted spectra. The dashed line represents the scaled 'total-sum' background. Bottom: Fully background-subtracted spectra normalized to the maximum C-H peak intensity at about 11 eV KE. The cross marks indicate the region used to determine the intensity of the C=O features.

References

1 J. Végh, *Journal of Electron Spectroscopy and Related Phenomena*, 2006, **151**, 159–164.

2 X. Li, Z. Zhang and V. E. Henrich, *Journal of Electron Spectroscopy and Related Phenomena*, 1993, **63**, 253–265.
